# Response to comment on “Pain in people living with HIV/AIDS: a systematic review (Parker *et al*. 2014)”

**DOI:** 10.7448/IAS.17.1.19234

**Published:** 2014-05-27

**Authors:** Romy Parker, Dan J Stein, Jennifer Jelsma

**Affiliations:** 1 Department of Health & Rehabilitation Sciences, Faculty of Health Sciences, University of Cape Town, Cape Town, South Africa; 2 Department of Psychiatry & Mental Health, Faculty of Health Sciences, University of Cape Town, Cape Town, South Africa

Dear editors,

We thank the authors of the Letter to the Editors for their thoughtful comments on our systematic review [1]. They emphasize a number of reasons why pain in people living with HIV/AIDS has clinical and health importance. We certainly agree with this view, and indeed these sorts of concerns motivated us to undertake the review.

The authors refer to four papers not included in the review [2–5]. Three of these papers were identified in the initial search but were excluded as they appeared to meet one of the exclusion criteria by reporting on sub-groupings of HIV-positive patients only [2,4,5]. While this criterion was arguably overly conservative, it is notable that the prevalence rates reported in these three papers fall within the range of estimates provided by the included studies. The fourth paper was published in early 2012 [3] and was not picked up by the literature search that we ran in March 2012. This paper is a useful contribution to the literature, and again the prevalence of pain falls within the estimates made in our review.

We would like to reiterate the point made by our colleagues in their letter; it is time for the research on pain in HIV/AIDS to move beyond identifying and describing the problem of pain in people living with HIV/AIDS and on to developing and testing interventions which will improve pain assessment and management.
